# Expert Consensus on Prevention and Treatment of Aging-Related Gonadal Dysfunction

**DOI:** 10.14336/AD.2024.0004

**Published:** 2024-04-10

**Authors:** Xi Wang, Li Ma, Sheng Jiang, Junping Wen, Zhaoli Yan, Long Tian, Shan Deng, Boxian Huang, Ilia Stambler, Calogero Caruso, Kyung-Jin Min, Huanxing Su, Kunlin Jin, Jiangfeng Mao, Xueyan Wu, Qin Han, Robert Chunhua Zhao

**Affiliations:** ^1^Peking Union Medical College Hospital, Beijing, China.; ^2^Traditional Chinese Medicine Hospital Affiliated to Xinjiang Medical University, Wulumuqi, China.; ^3^The First Affiliated Hospital of Xinjiang Medical University, Wulumuqi, China.; ^4^Fujian Provincial Hospital, Fuzhou, China.; ^5^Affiliated Hospital of Inner Mongolia Medical University, Huhehaote 010000, China.; ^6^Beijing Chao-Yang Hospital, Capital Medical University, Beijing 100020, China.; ^7^The affiliated Suzhou Hospital of Nanjing Medical University, Suzhou 215000, China.; ^8^Department of Science, Technology and Society, Bar-Ilan University, Ramat Gan, Israel.; ^9^Institute of Basic Medical Sciences, Chinese Academy of Medical Sciences, School of Basic Medicine, Peking Union Medical College, Beijing, China.; ^10^Growth and Development and Gonadal Diseases Committee of Chines Aging Well Association, China.; ^11^International Society on Aging and Disease, Bryan, TX, USA.; ^12^Department of Biomedicine, Neuroscience and Advanced Diagnostics, University of Palermo, Palermo, Italy.; ^13^Department of Biological Sciences, Inha University, Incheon, Republic of Korea.; ^14^Institute of Chinese Medical Science, University of Macau, Taipa, Macau, China.; ^15^University of North Texas Health Science Center, Bryan, TX, USA.; ^16^ School of Life Sciences, Shanghai University, shanghai, China.

**Keywords:** late-onset hypogonadism, erectile dysfunction, premature ovarian insufficiency, menopause, stem cell therapy, anti-aging

## Abstract

Aging-related hypogonadism involves complex mechanisms in humans, predominantly relating to the decline of multiple hormones and senile gonads. Late-onset hypogonadism (LOH) and erectile dysfunction (ED) are the main manifestations in men, while premature ovarian insufficiency (POI) and menopause are the main forms in women. Anti-aging measures include lifestyle modification and resistance training, hormonal supplementation, stem cell therapy, metformin, and rapamycin. In this expert consensus, the mechanisms, efficacy, and side effects of stem cell therapy on aging gonadal function are reviewed. Furthermore, various methods of stem cell therapy, administered intravenously, intracavernously, and intra-ovarially, are exemplified in detail. More clinical trials on aging-related gonadal dysfunction are required to solidify the foundation of this topic.

## 1. Decline in Gonadal Function with Age Impairs Quality of Life

Aging-related gonadal dysfunction is defined as the decline in gonadal function with advancing age, which has a profound impact on the quality of life in middle-aged and elderly individuals [1]. In females, a sharp decline in ovarian function leads to the presentation of menopausal syndrome [2], followed by a series of age-related conditions such as skin aging, reduced libido, increased cardiovascular disease risk, osteoporosis, and osteoarthritis. In males, the decline in testicular function is referred to as LOH. Symptoms of LOH are insidious and characterized by decreased physical performance, loss of muscle mass, diminished libido, ED, and sleep and mood disturbances [3]. These symptoms are all associated with aging. Currently, sex hormone supplementation constitutes the dominant intervention in clinical practice, but its efficacy is not satisfactory for some patients and to some degree. Comprehensive health guidance is employed, encompassing lifestyle modifications, resistance training to increase muscle mass, hormonal supplementation, and stem cell therapy [1]. These measures could attenuate the aging process, enhance muscle strength, and improve quality of life. Given these challenges, it is imperative to convene experienced clinical practitioners and multidisciplinary scientists to review the evidence for pharmaceutical interventions and provide recommendations for the prevention and treatment of aging-related gonadal dysfunction in clinical practice [4].

## 2. Menopause in Females

Menopause represents a pivotal juncture in the lives of females. The decline in estradiol levels due to ovarian insufficiency leads to a series of clinical manifestations, including hot flashes, irritability, anxiety, sweating, insomnia, discomfort during sexual intercourse, and disturbances in blood pressure stability [2,5]. Subsequently, the prolonged deficiency of estrogen results in the emergence of coronary heart disease and osteoporosis. The essence of menopause lies in the aging of ovaries [6].

When remarkable declines in sex hormone levels are identified and various contraindications are excluded, supplementation with estrogen may effectively delay the aging process. Estrogen supplementation significantly improves menopausal symptoms, reduces cardiovascular disease risk [7], enhances lipid profiles, and increases bone density [8]. However, patients at high risk for estrogen-dependent cancers, such as breast and endometrial cancer, are not suitable for hormonal replacement therapy.

## 3. LOH in Males

LOH, also referred to as andropause or Partial Androgen Deficiency in the Aging Male (PADAM), encompasses the essential nature of declining testosterone levels and waning functions in males as they age [3]. Despite the different terminology, they all reflect the gradual decrease in testosterone levels and the weakening of physiological functions in aging males. LOH primarily manifests as decreased physical performance, reduced muscle mass, diminished libido, ED, sleep disturbances, and mood changes. Doctors initially attributed these symptoms predominantly to decreased testosterone levels and believed that testosterone supplementation could alleviate these clinical manifestations [9]. Testosterone supplementation may improve symptoms of reduced libido and ED, improve body muscular composition, mitigate fatty liver, enhance glucose and lipid metabolism, and ameliorate glycemic control.

However, clinical studies have discovered that lowering levels of testosterone could not fully explain the complexity of the disease. First, a proportion of patients do not exhibit marked reductions in testosterone levels despite their pronounced obvious aging-related symptoms. Second, testosterone supplementation only improves symptoms in one-third of patients, and no significant effects were observed in the other two-thirds of cases. As a result, it is postulated that the clinical presentations of LOH should be attributed to various age-related hormone deficiencies (including growth hormone and adrenal-derived sex hormones) or be attributed to functional declines of multiple organs [10].

## 4. Systemic Nature of Aging

The functional decline in cells, tissues, and organs that occur with chronological age is referred to as aging [11]. Various systems exhibit distinct manifestations of aging, including decreased sex hormone levels, atherosclerosis, neurodegenerative changes, osteoporosis and osteoarthritis, weakened muscle mass, increased adipose deposition, altered T-cell function and subsets, increasing cancer risk, and a rise in chronic diseases such as diabetes and hypertension [11-13].

In clinical scenarios, the aging changes across multiple systems are treated by different specialties. For instance, heart diseases are managed by cardiologists, male gonadal dysfunction is managed by urologists, menopausal issues are addressed by gynecologists, and diabetes is treated by endocrinologists. However, aging is a systemic alteration with intricate interconnections between various systems. For example, the deficiency of estrogen directly leads to osteoporosis, subsequently increasing the risk of hip fractures and significantly compromising quality of life [11]. Therefore, it is imperative to explore anti-aging measures from a holistic perspective, aiming to fundamentally reverse or decelerate the aging process.

## 5. Potential of Stem Cell Therapy

**Figure 1.**
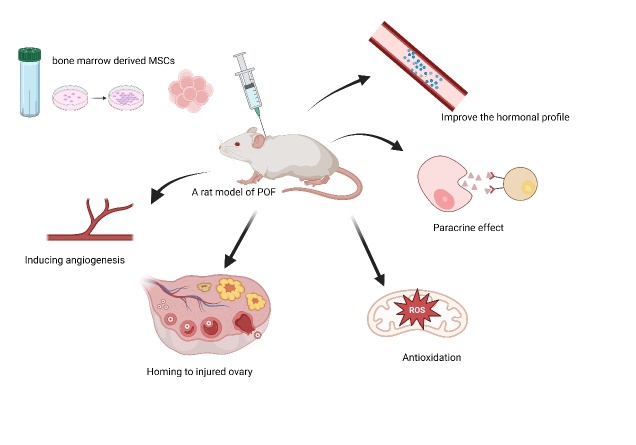
**The possible mechanisms of BMSCs in the treatment of POF**. BMSCs can improve ovarian function by inducing angiogenesis, homing to the injured ovary, exerting anti-oxidative stress, producing paracrine effects, and improving the hormonal profile.

**Figure 2.**
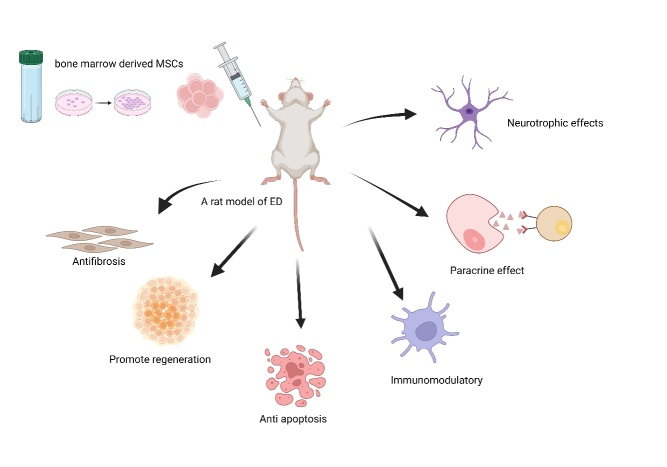
**The possible mechanisms of BMSCs in the treatment of ED**. BMSCs can improve ED by anti-fibrosis, promoting regeneration, anti-apoptosis, immunomodulation, paracrine effects, and neurotrophic effects on the cavernous nerves.

Stem cells introduced into the body exert anti-aging effects through various mechanisms: (1) They secrete anti-inflammatory factors, which can modulate the immune system, alleviate systemic inflammation, and improve immune-related biomarkers. Emerging evidence demonstrates that stem cell therapy can delay disease progression in COVID-19 patients, reduce the incidence of severe pneumonia, and accelerate recovery. (2) Stem cells can activate immune T cells, which can spot and clear aging cells. (3) Stem cells could enhance the ability to clear free radicals and exhibit antioxidative properties. (4) Pluripotent stem cells can further differentiate into specific tissue cells, replacing aging cells and enhancing organ function. (5) They promote cell proliferation and differentiation by secreting growth factors like insulin-like growth factor 1 (IGF-1). Substantial basic and clinical data support these mechanisms [14] (Fig. 1 and Fig. 2).

Mesenchymal stem cell (MSC) transplantation (administered intravenously) has demonstrated a significant anti-aging effect. Currently, over 200 clinical trials related to stem cells have been registered and are ongoing, covering diseases such as biliary cirrhosis, senile dementia, cerebral infarction, myocardial infarction, and retinal diseases. Many of these studies focus on age-related degenerative conditions such as cerebral infarction and type 2 diabetes [14]. Some research findings have shown promising therapeutic effects.

The mechanisms and effectiveness of stem cell transplantation in improving female ovarian function have become a hot topic in the gynecological field. Animal experiments have shown that MSCs or exosomes can effectively improve ovarian endocrine function and reproductive capacity [15]. MSCs maintain and restore ovarian function through various receptors and multiple signaling pathways. Through the mechanisms of cell homing, angiogenesis, immune regulation, and antioxidative stress pathways, MSCs may improve the function of various cell components, such as granulosa cells and primordial germ cells. Although they exhibited beneficial effects in animal models with premature ovarian failure (POF), clinical effectiveness requires further clarification. Numerous prospective studies are currently underway.

**Table 1:** The Effects of MSC Therapy on Ovarian Function.

No.	Diseases	N	Stem Cells	Transplantation Route	Efficacy	Time	References
**1**	Tubal occlusion (n=34) and POI (n=41)	75	hADSC-Exos	Intravenously	hADSC-Exos improve ovarian function by releasing exosomes through regulation of the SMAD pathway	2015	Huang, et al._2018^[16]^
**2**	POF	2	Autologous BMSCs	Intraovarian	Increases estrogen production and reduces menopausal symptoms	2016	Igboeli, et al_2020^[17]^ http://www.Clinicaltrials.gov, (NCT02696889)
**3**	POF	9	ADSCs	Intraovarian	Safe and feasible. Decreases FSH and resumes menstruation for more than 1 year.	2015	Mashayekhi _2021^[18]^ http://www.Clinicaltrials.gov (NCT02603744)
**4**	POF	10	MSC	Intraovarian	One case resumed menstruation after 3 months of therapy. Two cases with atrophic endometrium restored to focal secretory changes after therapy.	NR	Edessy, et al. 2014^[19]^
**5**	POF	10	MSC	Intraovarian	Improves hormonal profile. Regains menstruation. Achieves pregnancy and delivers a mature living healthy baby.	2014	Edessy, et al. 2016 ^[20]^
**6**	POF	60	AMSCs	Intraovarian	No results available.	2012	NCT02062931
**7**	DOR	12	hAMSCs	Intravenous	No results available.	2021	NCT04706312
**8**	POF	10	Autologous BMSCs	Intravenous	No results available.	2016	NCT02779374

POI, premature ovarian insufficiency; MSCs, mesenchymal stem cells; POF, premature ovarian failure; ADSCs, autologous adipose-derived stromal cells; FSH, follicle-stimulating hormone; Exos, exosomes; NR, not reported; AMSCs, autologous mesenchymal stem cells; BMSCs, bone marrow stem cells; DOR, diminished ovarian reserve; hAMSCs, human amniotic mesenchymal stem cells.

### (1)MSC Therapy for Ovarian Aging

**Figure 3.**
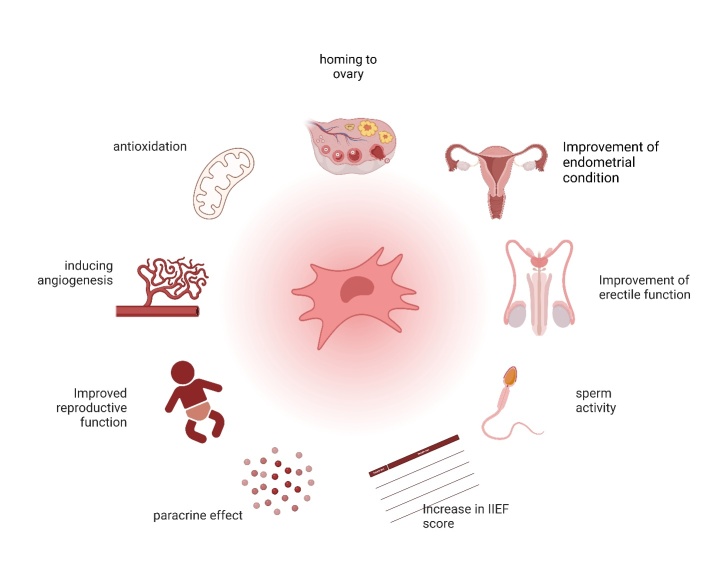
**The possible mechanisms and clear outcomes of mesenchymal stem cells (MSCs) in the treatment of POF and ED**. MSCs improve POF-induced hypogonadism and ED through the mechanisms of anti-oxidation, inducing angiogenesis, and paracrine effects. Clinical outcomes suggest that MSCs improve endometrium and reproductive function in females, and erectile function (IIEF scores) and sperm activity in males.

Clinical trials related to stem cell therapy for patients with POI and menopause have demonstrated some beneficial effects on ovarian function. Different types of stem cells were adopted in clinical trials, such as bone marrow-derived MSCs (BMSCs), autologous adipose-derived stromal cells (ADSCs), and human amniotic MSCs (hAMSCs). Various methods were adopted for stem cell transplantation, such as intraovarian injection, intravenous injection, and ovarian artery injection (Fig. 3). These trials are listed in Table 1.

Here is a reference for MSC therapy applied to patients with POI: (1) Preparation of autologous BMSCs: Under general anesthesia, bone marrow tissue was collected from the posterior iliac crest using a bone marrow aspiration kit, resulting in the extraction of approximately 150 mL of marrow tissue. Subsequently, the harvested tissue is centrifuged, and the cell pellet, comprising an estimated total of 5×108 nucleated cells and an average of 5-15 million MSCs, is obtained. (2) The harvested BMSCs (4 mL) were directly injected into the ovary using a 5-mm injector equipped with a 22-gauge needle by laparoscopy. An assistant stabilized the ovary with an atraumatic grasper, and the surgeon inserted the needle through a single point in the ovarian capsule towards the center of the ovary. The MSCs were then administered slowly over a duration of 5 minutes, allowing for the gradual expansion of the ovary. After complete administration, the needle remained in place for an additional 5 minutes, serving to prevent any potential backflow of injected cells. (3) After MSC engraftment, a standard transvaginal ultrasound technique was employed to measure endometrial thickness, ovarian volume, and antral follicle count (AFC) at 1, 3, 6, 9, and 12 months. Serum hormone profiles were monitored post-engraftment at intervals of the same period. The assessed hormones encompassed follicle-stimulating hormone (FSH), luteinizing hormone (LH), anti-Müllerian hormone (AMH), estradiol (E2), and progesterone. Symptoms of menopause should be evaluated after MSC therapy.

Here is another example of hAMSC therapy applied to women with diminished ovarian reserve (DOR) by an intravenous route:(1) The hAMSCs were isolated, cultured in vitro, and subsequently validated by the National Institutes for Food and Drug Control in China. (2) The serum of each patient was collected and sent for laboratory testing prior to the transplantation. The biomarkers of hAMSCs were re-evaluated before the transplantation. The hAMSCs were then administered intravenously into the dorsal vein of the hand. (3) After hAMSC engraftment, sex hormonal levels, FSH, LH, AMH, and endometrial thickness were evaluated at 3, 6, 9, and 12 months.

### (1)MSC Therapy for Males with LOH and ED

Clinical trials have indicated that, overall, MSC therapy could lead to improvements in ED (Fig. 3). The specific outcomes of these trials are listed in Table 2. However, it is worth noting that none of the results demonstrated a significant improvement in testosterone concentration as a result of MSC therapy.

Here is an example of MSC transplantation for patients with ED:(1) Dexamethasone 2 mg is administered intravenously before umbilical cord-derived MSC (UC-MSC) injection. Then, UC-MSCs (2-4 ×10^7^ MSCs dissolved in 100 mL of 0.9% saline solution) are intravenously injected. The filter screen in the infusion device should be removed before stem cell injection. Two weeks later, another bolus of UC-MSCs (2-4 ×10^7^ MSCs) is administered intravenously again. (2) After infusion, UC-MSCs may lodge in pulmonary microvessels, potentially leading to microembolisms. Therefore, vital signs should be monitored during the transplantation procedure. (3) Patients were regularly followed up at 30, 90, and 180 days after UC-MSC therapy. Erectile function, quality of life, and sleep status could be assessed using the International Index of Erectile Function (IIEF) scale, the 36-Item Short Form Survey (SF-36) scale, and sleep questionnaires. Age-related hormones, including FSH, LH, testosterone, and IGF-1, could be evaluated.

**Table 2:** The Effects of MSC Therapy for Male LOH and ED.

No.	Diseases	N	Stem Cell Line	Transplantation Route	Efficacy	Clinical Trial	Reference
**1**	Post-radical prostatectomy	21	ADRCs	Intracavernous	53% of erections are sufficient for intercourse. Not seen in incontinent or pre-op ED men.	12 months, Open label phase I	Haahr, et al. 2018 ^[21]^ NCT02240823
**2**	ED	8	Placental derived MSCs	Intracavernous	Increase in peak systolic velocity at 6 months. Three patients had the ability to sustain erections on their own.	Open label phase I	Levy, et al. ^[22]^ NCT02398370
**3**	Post Prostatectomy ED	6	BM-MNCs	Intracavernous	Improvement in two IIEF categories (intercourse satisfaction, and erectile function) after 6 months.	Phase I: 2016 Phase II: 2017	Yiou, et al. ^[23]^ NCT01089387
**4**	ED	40	Autologous BM	Intracavernous	Improvement in IIEF scores at 6 months.	NR	Bieri, et al. ^[24]^ NCT03699943
**5**	Post Prostatectomy ED	10	BM-MNCs	Intracavernous	Improvement in IIEF scores at 1 month.	NR	You, et al. ^[25]^ NCT02344849
**6**	DM and ED	4	BM-MNCs	Intracavernous	Good tolerance to procedure. Improvements in IIEF-15 and EHS	Open label phase I	Al Demour, et al. ^[26]^ NCT02945462
**7**	DM and ED	10	UC-MSC	Intracavernous	Improved morning erections at 3 months. 2/7 patients had sufficient erection with PDE-5 inhibitor at 6 months.	Single-blind phase I	Bahk, et al. ^[27]^ NR

LOH, late-onset hypogonadism; ED, erectile dysfunction; ADRCs, adipose-derived regenerative cells; MSCs, mesenchymal stem cells; DM, diabetes mellitus; IIEF, International Index of Erectile Function; EHS, Erection Hardness Score; UC-MSCs, umbilical cord-derived mesenchymal stem cells; PDE-5, phosphodiesterase type 5; NR, not reported.

Here is another option for autologous BMSC transplantation by direct intracavernosal injection for patients with ED:(1) Preparation of autologous BMSCs involves obtaining approximately 10 mL of bone marrow from the posterior superior iliac crest. Following density gradient centrifugation and a wash with phosphate-buffered saline, the cells are separated, resuspended, and cultured. (2) BMSCs are slowly injected into the corpus cavernosum on both sides. The harvested BMSCs (2 mL, containing 3×10^7^ autologous BMSCs) are administered into the corpus cavernosum using a prefilled syringe equipped with a 21-gauge needle. To ensure an even distribution of stem cells throughout the corpus cavernosum, a tourniquet is applied at the base of the penis to induce an artificial erection.(3) After monitoring for adverse events for 2 hours post-injection, patients are allowed to return home.(4) Efficacy outcome measures encompass changes in the IIEF score, Sexual Encounter Profile (SEP) questions 2 and 3, Global Assessment Question (GAQ) scores at 1, 3, 6, 9, and 12 months, as well as penile Doppler sonography (PDS) at 6 and 12 months after the administration of autologous BMSCs.

## 6. Other Approaches in Clinical Practice

Other multifaceted approaches should be used to counteract and ameliorate the age-related decline in gonadal function ^[4]^, including supplementation of growth hormone, comprehensive lifestyle modification, and medication with metformin and rapamycin.

(1) Supplementation of Growth Hormone: For patients with growth hormone deficiency, supplementing with physiological doses of recombinant human growth hormone can distinctly increase muscle mass, improve body composition, reverse hepatic adiposity, improve skin quality, and ultimately enhance quality of life ^[28]^.

(2) Comprehensive Lifestyle Modification: Comprehensive lifestyle modifications encompass several aspects: reducing sedentary time and encouraging aerobic exercises, adopting a diet primarily containing vegetables and fruits, and advocating for appropriate dietary control to reduce the risk of obesity ^[29]^. Evidence suggests that meditation is a useful practice to enhance cognitive abilities ^[30]^ and promote overall well-being.

Recent evidence demonstrates that engaging in aerobic exercises for at least 30 minutes, three times a week, contributes to improved cardiovascular status ^[31]^. Resistance exercise, involving appropriate weights and strength training, is also effective in enhancing muscle strength [32].

(3) Metformin: Metformin is a classic anti-glycemic medication [33]. Multiple animal and clinical studies have shown that metformin can extend the lifespan of mice and humans. Patients taking metformin exhibit a 15% additional increment in lifespan compared to those who do not [28]. The risk of various tumors, such as pancreatic cancer, is also significantly reduced.

The anti-aging mechanism of metformin is associated with the activation of the adenosine monophosphate-activated protein kinase (AMPK) signaling pathway and induction of autophagy. It alleviates aging characteristics through mechanisms such as the improvement of nutrient sensing (insulin/IGF-1 signaling), enhancing intercellular communication, stabilizing protein homeostasis, maintaining genomic stability, regulating mitochondrial function, preventing macromolecular damage, delaying stem cell aging, modulating transcription, and reducing telomere attrition [34].

(4) Rapamycin: Rapamycin acts by inhibiting the mammalian target of rapamycin (mTOR) signaling pathway, activating autophagy, and restoring cellular vitality. Rapamycin can extend lifespan, improve health biomarkers, and reduce the risk of some chronic diseases, including cancer, cardiovascular disease, neurodegenerative diseases, obesity, and type 2 diabetes [35].

## 7. Future Directions in Anti-Aging Treatments

In the future, novel medications encompass two main strategies: reversing aging cells to restore their normal functionality or promoting aging cells to undergo apoptosis and reducing their detrimental effects on surrounding cells and tissues. These foundational studies are guiding the direction of future clinical applications.

New biomarkers for evaluating the progression of aging should be established. Aging is a process that can be quantitatively evaluated using specific biomarkers. For humans, aging is manifested as reduced physical performance (evaluated by decreased oxygen consumption), decreased sexual function (evaluated by erectile function scales), increased fat proportion, decreased lean body mass (assessed via computed tomography [CT] or magnetic resonance [MR] imaging), ectopic fat accumulation (fat in skeletal muscle, liver, thymus), insomnia (sleep quality scales), impaired memory, cognitive function, and quality of life (SF-36 health survey). Aging-related biomarkers include interleukin-6 (IL-6), IL-10, tumor necrosis factor-alpha (TNF-α), high-sensitivity C-reactive protein (hsCRP), T-cell subsets, sex hormones, IGF-1, and adrenal-derived hormones (sulfated dehydroepiandrosterone and dihydrotestosterone).

In the future, the efficacy of medications and stem cell therapy can be evaluated by both subjective scales and objective biomarkers [36]. The improvement in symptoms after stem cell therapy can be evaluated using simple questionnaires, including the Istanbul Male Menopause Symptom Score, the IIEF, the SF-36 Health Survey, and the Self-Rating Sleep Status (SRSS). For patients undergoing long-term anti-aging treatment, evaluations can also be conducted using indices such as body fat percentage (measured by dual-energy X-ray absorptiometry [DXA]), bone mineral density (measured by DXA), and intrahepatic fat content (measured by MR imaging).

## 8. Conclusion

Hypogonadism represents one facet of the multiple systemic manifestations of aging. For women, menopause marks a pivotal transition from middle to old age. There is an increasing incidence of cardiovascular events and osteoporosis after menopause. Similar patterns emerge in men. As aging constitutes a synchronized progression across multiple systems throughout the body, novel techniques and approaches should be considered to systematically prevent the aging progression and improve overall health status. Treatment modalities encompass lifestyle modification, hormonal supplementation, stem cell therapy, and other anti-aging medications. This expert consensus serves as a standardized reference for the prevention and management of age-related hypogonadism and systemic aging. Simultaneously, multicenter, prospective, and randomized clinical trials on anti-aging are encouraged to provide more evidence-based medical insights into delaying the aging process and improving health quality. Guidelines for the prevention and treatment of this topic will be continuously updated with the emergence of additional evidence.
